# Morphometric analysis of safe working zones for minimally invasive lateral extrapleural/retroperitoneal approach via transdiaphragmatic and transpsoas to the thoracolumbar and lumbar spine

**DOI:** 10.1186/s13018-025-06219-8

**Published:** 2025-09-30

**Authors:** Zhengming Lv, Jian Tong, Haijun Li, Yongxin Ren

**Affiliations:** 1 Department of orthopedics, Taizhou School of Clinical Medicine, The Affiliated Taizhou people’s Hospital of Nanjing Medical University, Nanjing Medical University, 366 Taihu road, Taizhou, 225300 Jiangsu China; 2 Department of orthopedics, The First Affiliated Hospital of Nanjing Medical University, Jiangsu Province People’s Hospital, 300 Guangzhou Road, Nanjing, 210029 Jiangsu China

**Keywords:** Lateral extrapleural/Retroperitoneal approach, Transdiaphragmatic and transpsoas, Thoracolumbar and lumbar spine, Safe working zones

## Abstract

**Background:**

The thoracolumbar and lumbar spine is mainly involved in T11 to L5, which is the transition from the relatively fixed thoracic vertebrae to the more mobile lumbar vertebrae, where the main stress of the trunk is concentrated.This study aims to perform a detailed morphometric analysis of the thoracolumbar and lumbar region using high-resolution CT scans to define safe working zones for the transdiaphragmatic and transpsoas approaches.

**Methods:**

Patients who underwent CT examination from February 2024 through September 2024 were from our database. Measurements were performed from T10-T11,T11-T12,T12-L1,L1-L2,L2-L3,L3-L4 and L4-L5 disc levels and were determined using the PACS software computer digitizer and SYNGO System. Using this data, one surgeon carried out measurements of the lower vertebral endplate at each level (sagittal and transversal), position of muscle attachment area, position of the nerve roots, position of the retroperitoneal vessels.The safe zone is defined as the region between retroperitoneal vessels (including arteries and veins) and nerve roots. Its superior boundary is demarcated by the horizontal plane of the most inferior vessel border, while the inferior boundary corresponds to the horizontal plane of the most superior nerve root border.

**Results:**

In all subjects, the transverse diameters demonstrated a progressive increase across the T10–T11 to L4–L5 spinal segments. Sagittal diameters exhibited a similar ascending trend from T10–T11 to L2–L3, followed by a gradual reduction from L2–L3 to L4–L5. Notably, the smallest sagittal diameter was recorded at T10–T11, while the maximum value peaked at L2–L3.The projection of nerve roots is identical on both sides. The nerve-vertebral overlap height progressively decreased from T10/T11 to L1/L2, then progressively increased from L1/L2 to L4/L5, with the maximal overlap observed at L4/L5 and the minimal at L1/L2.The vascular-vertebral overlap height progressively decreased from T10/T11 to L1/L2, then progressively increased from L1/L2 to L4/L5, with the maximal overlap observed at T10/T11 and the minimal at L1/L2.No diaphragmatic attachments were observed bilaterally at the T10-T11 level. Partial bilateral diaphragmatic attachments with craniocaudal elevation were identified at T11/T12 in 28.3% of specimens(17/60), while the majority exhibited diaphragmatic attachments at T12/L1 in 98.3% of specimens(59/60).A progressive increase in the safety zone ratio was observed sequentially across the T10–T11, T11–T12, T12–L1, and L1–L2 spinal levels, reaching its maximum at L1–L2. Conversely, a gradual decrease occurred from L1–L2 to L2–L3, L3–L4, and L4–L5, with the L1–L2 level demonstrating the highest safety zone ratio.The safe working zone was 51.8% of the lower endplate of the vertebral body sagittal diameter at T10-T11, 63.9% at T11-T12, 73.5% at T12-L1,79.6% at L1–L2, 67.6% at L2–L3, 60.7% at L3–L4 and 48.6% at L4–L5 levels.

**Conclusion:**

Three-dimensional CT reconstruction enables clear visualization of vascular structures, vertebral bodies, muscle attachment sites, nerve roots, and their spatial relationships. This modality further allows precise preoperative delineation of discectomy and corpectomy boundaries via transdiaphragmatic and psoas muscle approaches, while quantitatively assessing safety working zones for surgical planning.

**Supplementary Information:**

The online version contains supplementary material available at 10.1186/s13018-025-06219-8.

## Background

The thoracolumbar and lumbar spine is an area of great interest for orthopedic surgeons [1]. It is mainly involved in T11 to L5, which is the transition from the relatively fixed thoracic vertebrae to the more mobile lumbar vertebrae, where the main stress of the trunk is concentrated [2, 3]. Due to its unique biomechanical properties, it is often the site of various pathological conditions, including trauma, degenerative diseases (such as disc herniation and spinal stenosis), infections, tumors, and deformities (such as scoliosis) [4, 5].

While traditional open surgical approaches are effective in managing these conditions, they are associated with significant complications and high morbidity. Open surgery typically requires extensive muscle dissection, leading to postoperative pain, prolonged recovery, and potential loss of muscle function [6, 7]. Additionally, open surgery may increase the risk of infection, bleeding, and nerve injury [8].

With the advancement of minimally invasive spine surgery (MISS), an increasing body of research has demonstrated that minimally invasive techniques can significantly reduce tissue trauma, shorten hospital stays, decrease postoperative pain, and accelerate patient recovery [9–11]. Among these techniques, the lateral extrapleural/retroperitoneal approach, accessed via transdiaphragmatic and transpsoas pathways, provides direct access to the thoracolumbar and lumbar spine while avoiding the extensive disruption of paraspinal muscles associated with traditional posterior approaches [1, 12, 13]. This approach is particularly suitable for anterior column pathologies of the thoracolumbar and lumbar spine, such as vertebral fractures, degenerative disc disease, and tumor resection [14, 15].

However, despite the significant advantages of minimally invasive techniques, their technical complexity presents new challenges.The anatomical structures involved in the transdiaphragmatic and transpsoas pathways are complex and include critical structures such as the diaphragm, psoas muscle, and neurovascular bundles (e.g., lumbar plexus, aorta, and inferior vena cava) [16, 17]. Injury to these structures can lead to severe complications, including neurological deficits, vascular injury, and damage to abdominal organs [18, 19]. Therefore, defining safe working zones is crucial for minimizing surgical risks.

In recent years, several studies have attempted to provide anatomical guidance for the lateral approach through imaging and anatomical analyses. For example, Uribe et al. [20] defined the distribution of the lumbar plexus within the psoas muscle through cadaveric studies, offering important anatomical references for the transpsoas approach. However, detailed morphometric analyses of the thoracolumbar and lumbar region remain limited, particularly regarding the complex anatomical relationships involving the diaphragm and retroperitoneal space [19]. Furthermore, existing research has predominantly focused on the lumbar region, with fewer studies addressing the transitional thoracolumbar area [21, 22].

This study aims to perform a detailed morphometric analysis of the thoracolumbar and lumbar region using high-resolution CT scans to define safe working zones for the transdiaphragmatic and transpsoas approaches. By quantifying the spatial relationships of critical structures, this study will provide surgeons with precise anatomical guidance to optimize the safety and efficacy of these surgical techniques.

## Methods

Patients who underwent CT examination from February 2024 through September 2024 were from our database. The CT scans were performed using a 128-detector row, 256-slice scanner (SOMATOM Definition Flash; Siemens Healthineers, Germany). All participants underwent contrast-enhanced thoracoabdominal CT examinations. Inclusion criteria were as follows: clear visualization of thethoracolumbar and lumbar spine (T10-L5 segments); availability of complete imaging data for three-dimensional reconstruction; and absence of thoracolumbar and lumbar abnormalities, diseases, trauma, or surgical history. Exclusion criteria included: spinal pathologies (e.g., scoliosis, spondylolisthesis, vertebral fractures, disc degeneration); history of thoracolumbar and lumbar tuberculosis or tumors; prior surgery; long-term use of hormonal medications; systemic conditions such as ankylosing spondylitis and rheumatoid arthritis.We retrospectively evaluated thoracolumbar and lumbar spine CT in 60 patients (mean age: 29.9 years, ranging from 21 to 39, SD: 5.8), 30 males (mean age: 30.3 years, ranging from 21 to 39, SD: 5.9), 30 females (mean age:29.7 years, ranging from21 to 39, SD: 5.9). The range of examined segments covered T10 through L5. The total number of thoracolumbar and lumbar vertebrae measured was 420 levels.

**Fig. 1:**
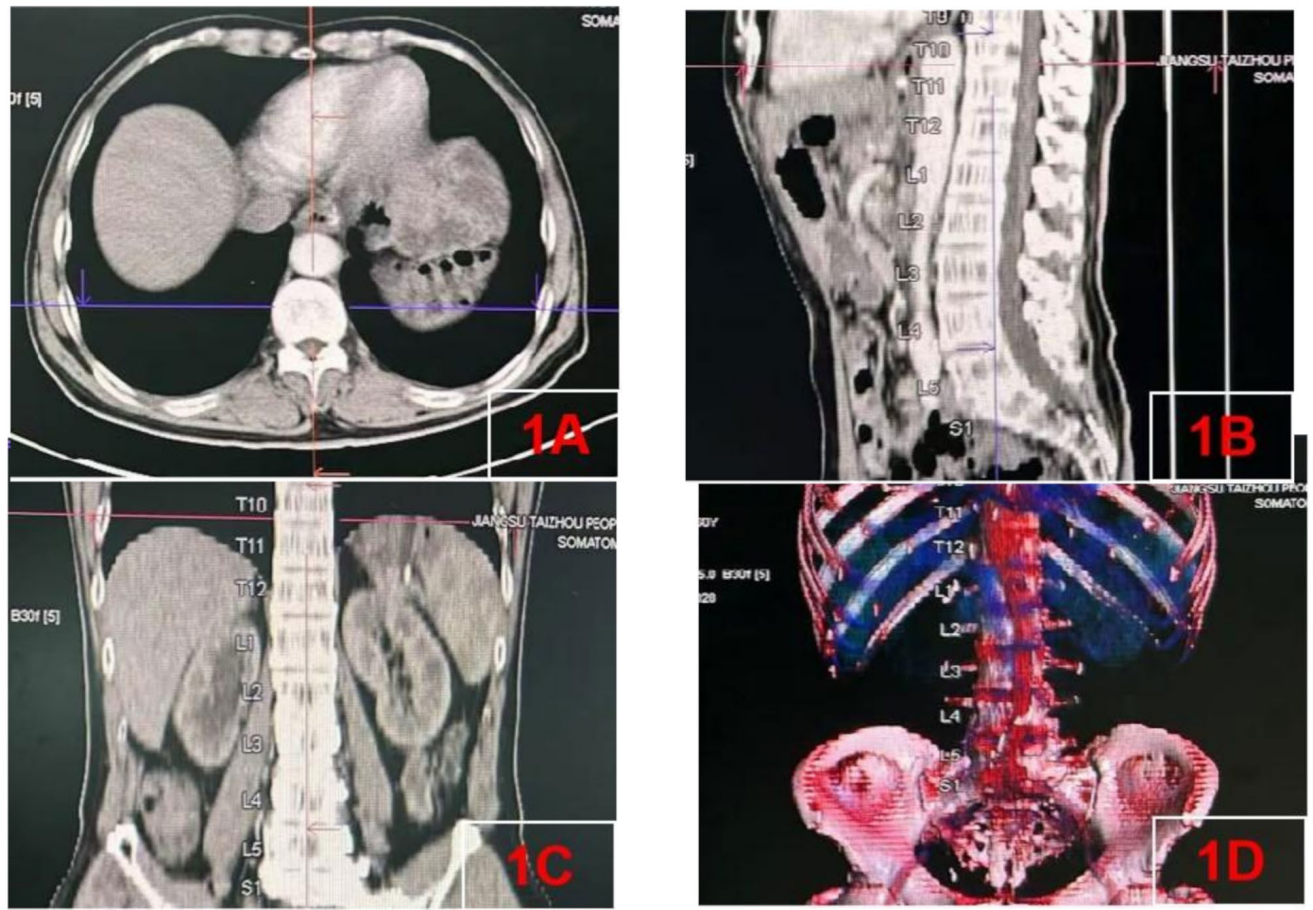
CT images: **A**, cross-sectional (axial) images of the thoracolumbar and lumbar spine; **B**, sagittal plane visualization of the thoracolumbar and lumbar region; **C**, coronal plane anatomical sections through the thoracolumbar and lumbar vertebrae; **D**, three-dimensional reconstructed volumetric rendering of the thoracolumbar and lumbar spine

Measurements were performed from T10-T11,T11-T12,T12-L1,L1-L2,L2-L3,L3-L4 and L4-L5 disc levels and were determined using the PACS software computer digitizer and SYNGO System (Siemens Healthineers, Germany). The CT image datasets were imported into PACS software computer digitizer and SYNGO System for three-dimensional (3D) reconstruction. Anatomical parameters in sagittal and axial planes were subsequently localized and quantified within the SYNGO system (Fig. 1).

**Fig. 2:**
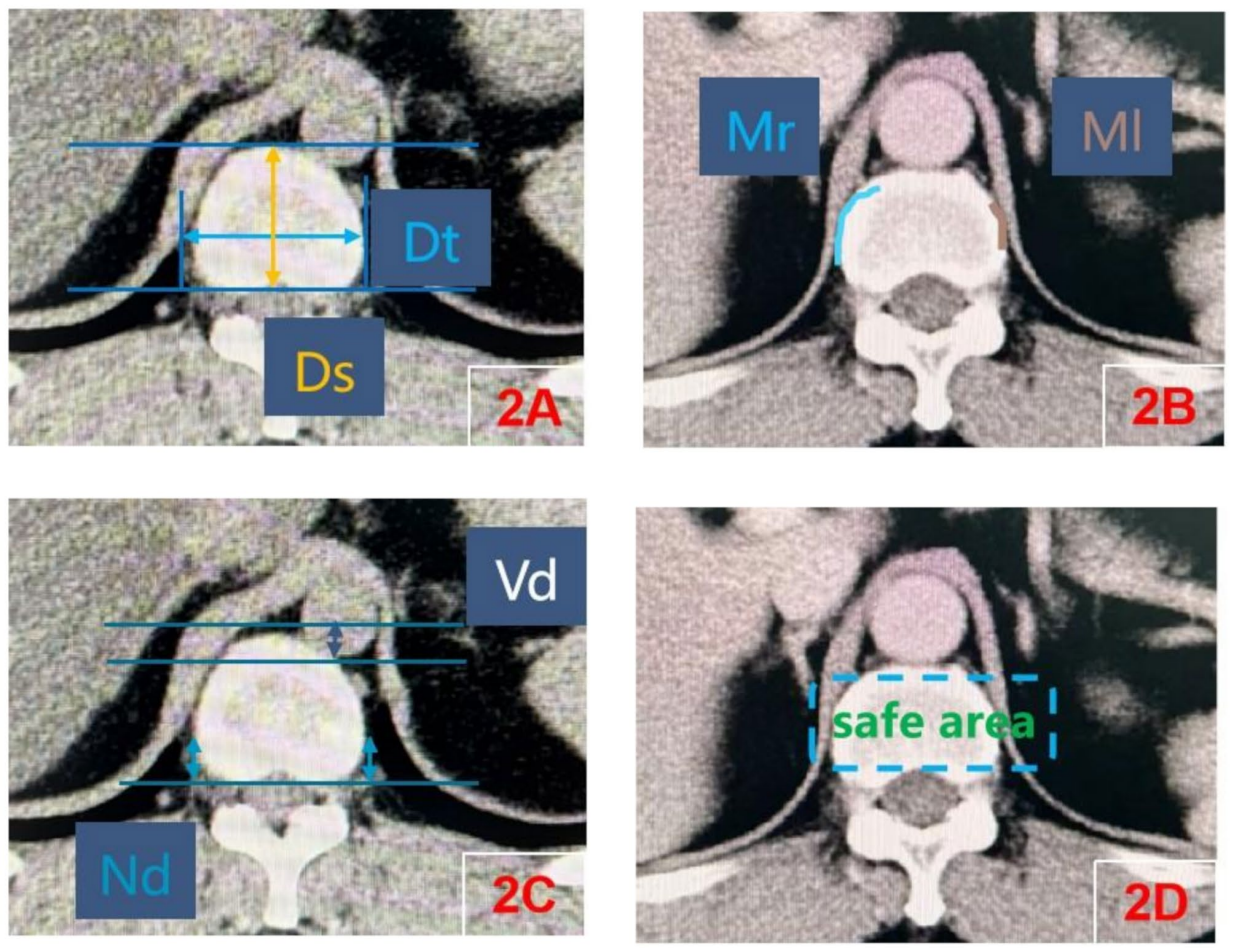
CT measurements: **A**, Measurement of vertebral endplate diameter(Dt transversal diameter, Ds sagittal diameter); **B**, Measurement of muscle attachment area(Mr the right muscle attachment area, Ml the left muscle attachment area); **C**, Measurement of vascular position(Vd) and nerve root position(Nd); **D**, Measurement of safe area

Using this data, one surgeon carried out measurements of the lower vertebral endplate at each level (sagittal and transversal), position of muscle attachment area, position of the nerve roots, position of the retroperitoneal vessels (Fig. 2A/B/C).

Extent measurements of the overlap of the retroperitoneal vessels and nerve roots with the lower endplate of vertebral body were performed from both sides of the spine. Measurements were obtained from the posterior or the anterior border of the lower endplate at each intervertebral disc level. Then, the extent of the overlap of the retroperitoneal vessels with the ventral edge of the lower vertebral body endplate was measured.The safe zone is defined as the region between retroperitoneal vessels (including arteries and veins) and nerve roots. Its superior boundary is demarcated by the horizontal plane of the most inferior vessel border, while the inferior boundary corresponds to the horizontal plane of the most superior nerve root border. (Fig. 2D).

### Statistics

All data were statistically analyzed using SPSS software 17.0 (SPSS Inc, Chicago, IL). Continuous variables conforming to a normal distribution were expressed as mean ± standard deviation (SD). Overall comparisons among groups were performed using randomized block analysis of variance (ANOVA), and pairwise comparisons were conducted with the least significant difference (LSD) test. For continuous variables violating normality, data were presented as median (interquartile range, IQR), with overall comparisons analyzed using non-parametric tests and pairwise comparisons assessed via the Nemenyi test. Categorical variables were described as frequency (percentage), and differences between groups were evaluated using the Chi-square test. A two-tailed *P* < 0.05 was considered statistically significant.

## Results

### Vertebral endplate diameter

**Fig. 3:**
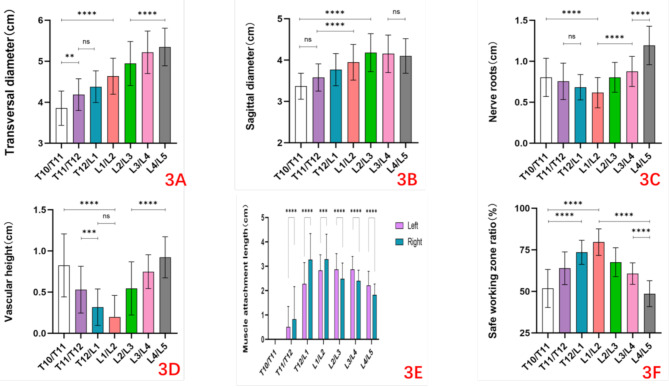
The statistical analysis results of vertebral and intervertebral disc parameters

The transverse diameters demonstrated a progressive increase across the T10–T11 to L4–L5 spinal segments (Table 1; Fig. 3A).Sagittal diameters exhibited a similar ascending trend from T10–T11 to L2–L3, followed by a gradual reduction from L2–L3 to L4–L5. Notably, the smallest sagittal diameter was recorded at T10–T11, while the maximum value peaked at L2–L3 (Table 1; Fig. 3B).

**Table 1:** The measured values of Vertebral transversal diameter and sagittal diameter

Variable	Level	Measured Values(cm)			
		Mean	Min	Max	SD
Vertebral transversal diameter	T10/T11	3.85	3.10	5.19	0.42
	T11/T12	4.19	3.51	5.20	0.39
	T12/L1	4.38	3.71	5.36	0.38
	L1/L2	4.64	3.78	5.81	0.44
	L2/L3	4.95	3.93	6.2	0.54
	L3/L4	5.22	4.06	6.82	0.22
	L4/L5	5.35	4.58	6.32	0.46
Vertebral sagittal diameter	T10/T11	3.67	2.78	4.13	0.32
	T11/T12	3.58	2.99	4.50	0.33
	T12/L1	3.77	3.01	4.59	0.39
	L1/L2	3.95	3.18	4.91	0.43
	L2/L3	4.18	3.39	5.35	0.46
	L3/L4	4.15	3.28	5.4	0.45
	L4/L5	4.10	3.25	5.6	0.42

### Position of the neuro-vascular structures

Data concerning the position of nerve roots, the position of retroperitoneal vessels are presented in Table 2. Extent measurements of the overlap of the retroperitoneal vessels and nerve roots with the lower endplate of vertebral body were performed from both sides of the spine. The projection of nerve roots is identical on both sides. The nerve-vertebral overlap height progressively decreased from T10/T11 to L1/L2, then progressively increased from L1/L2 to L4/L5, with the maximal overlap observed at L4/L5 and the minimal at L1/L2 (Table 2; Fig. 3C).

At the T10–T11 junction, vascular analysis revealed leftward displacement of the aorta, azygos, and hemiazygos veins in partial cases.The T11–T12 level demonstrated rightward transition of the thoracic aorta, predominantly positioned slightly leftward or directly anterior.Transitioning to T12–L1, the thoracic aorta maintained an anterior midline position. The vascular configuration transitioned to anterior midline orientation in lumbar regions, culminating in rightward branching patterns at L4–L5 with multiple terminal divisions. The vascular-vertebral overlap height progressively decreased from T10/T11 to L1/L2, then progressively increased from L1/L2 to L4/L5, with the maximal overlap observed at T10/T11 and the minimal at L1/L2 (Table 2; Fig. 3D).

**Table 2:** The measured values of Nerve root height and Retroperitoneal vascular height.The measured values of the muscle attachment lengths on the left and right sides

Variable	Level	Measured Values(cm)			
		Mean	Min	Max	SD
Nerve root height	T10/T11	0.80	0.47	1.83	0.23
	T11/T12	0.76	0.44	1.92	0.22
	T12/L1	0.68	0.4	1	0.16
	L1/L2	0.62	0.3	1.13	0.18
	L2/L3	0.80	0.44	1.25	0.18
	L3/L4	0.88	0.51	1.4	0.18
	L4/L5	1.19	0.67	1.7	0.23
Retroperitoneal vascular height	T10/T11	0.82	0.32	1.98	0.38
	T11/T12	0.53	0	1.4	0.28
	T12/L1	0.32	0	0.78	0.22
	L1/L2	0.20	0	1	0.26
	L2/L3	0.55	0	1.35	0.32
	L3/L4	0.75	0.36	1.4	0.21
	L4/L5	0.92	0.5	1.73	0.25

### Muscle attachment area

No diaphragmatic attachments were observed bilaterally at the T10-T11 level. Partial bilateral diaphragmatic attachments with craniocaudal elevation were identified at T11/T12 in 28.3% of specimens(17/60), while the majority exhibited diaphragmatic attachments at T12/L1 in 98.3% of specimens(59/60).Characteristic bilateral distribution patterns were demonstrated: right-sided attachments occupied anterolateral positions, whereas left-sided attachments localized laterally, partially overlapping vascular structures. Right-sided diaphragmatic attachments showed greater spatial extent than the left at both T11/T12 and T12/L1 levels. Caudal transition of diaphragmatic fibers into the psoas major was observed in lumbar regions. The psoas major exhibited symmetrical bilateral attachments along vertebral laterality, with reduced right-sided attachment areas at L2/L3, L3/L4, and L4/L5 compared to the left. In Table 3; Fig. 3E.

**Table 3:** The measured values of the muscle attachment lengths on the left and right sides

Variable	Level	Measured Values(cm)			
		Mean	Min	Max	SD
Left muscle attachment length	T10/T11	0	0	0	0
	T11/T12	0.51	0	2.63	0.84
	T12/L1	2.28	0	4.18	0.87
	L1/L2	2.83	1.52	4.43	0.65
	L2/L3	2.87	1.55	5.02	0.65
	L3/L4	2.88	1.69	4.19	0.53
	L4/L5	2.21	1.32	4.37	0.58
Right muscle attachment length	T10/T11	0	0	0	0
	T11/T12	0.82	0	3.58	1.34
	T12/L1	3.27	0	5.18	1.07
	L1/L2	3.29	1.49	5.73	1.03
	L2/L3	2.49	1.38	4.68	0.65
	L3/L4	2.4	1.47	3.22	0.44
	L4/L5	1.83	1.1	2.91	0.44

### Safe working zones

In all subjects, a progressive increase in the safety zone ratio was observed sequentially across the T10–T11, T11–T12, T12–L1, and L1–L2 spinal levels, reaching its maximum at L1–L2. Conversely, a gradual decrease occurred from L1–L2 to L2–L3, L3–L4, and L4–L5, with the L1–L2 level demonstrating the highest safety zone ratio.The safe working zone was 51.8% of the lower endplate of the vertebral body sagittal diameter at T10-T11, 63.9% at T11-T12, 73.5% at T12-L1,79.6% at L1–L2, 67.6% at L2–L3, 60.7% at L3–L4 and 48.6% at L4–L5 levels in Table 4; Fig. 3F.

**Table 4:** The parameters of safe working area ratio

Variable	Level	Measured Values(%)			
		Mean	Min	Max	SD
Safe working zone ratio	T10/T11	51.8	24.9	69.6	11.5
	T11/T12	63.9	30.3	79.3	9.9
	T12/L1	73.5	56.9	89.7	7.2
	L1/L2	79.6	58.8	89.8	8.0
	L2/L3	67.6	51.0	85.3	8.7
	L3/L4	60.7	45.4	73.9	6.5
	L4/L5	48.6	28.5	63.2	7.8

## Discussions

With the advancement of minimally invasive techniques and the development of surgical instruments, many spinal surgeries now emphasize minimizing tissue damage and bleeding. The lateral minimally invasive small incision technique has been increasingly applied in clinical practice, emerging as a new feasible approach for treating thoracolumbar and lumbar anterior column pathologies [8, 23]. Minimally invasive extrapleural retroperitoneal approach is a novel surgical access that allows for discectomy and corpectomy procedures [23, 11]. The anterior surgery allows direct access to the lesion site, but may cause the manipulation and potential injury to the retroperitoneal vessels and abdominal organs [24]. While the posterior approach does not allow for direct decompression of the anterior and middle spinal columns, the anterolateral surgical approach enables targeted resection of pathological foci with preservation of posterior column integrity. However, this technique is associated with potential complications, including vascular injury and retrograde ejaculation [25, 26].

The lateral approach presents three principal intraoperative challenges: (1) precise quantification of osteotomy dimensions (depth/width) under limited visual fields, (2) avoidance of iatrogenic trauma to ipsilateral/contralateral great vessels (e.g., abdominal aorta/vena cava) and segmental nerve roots, and (3) reliable identification of diaphragmatic attachment zones spanning thoracolumbar junctions.

From a lateral perspective, the transverse diameter of the vertebral body represents the surgical depth, while the sagittal diameter corresponds to the surgical width. This study observed that the transverse diameter gradually increases from T10–T11 to L4–L5, indicating a progressive increase in surgical depth. Meanwhile, the sagittal diameter progressively enlarges from T10–T11 to L2–L3 but decreases from L2–L3 to L4–L5, reflecting a corresponding trend in surgical width. For preoperative planning, the range of surgical parameters should be determined by referencing measurements from adjacent vertebral levels.

Current research on the diaphragm predominantly focuses on cadaveric dissection studies, with limited exploration in imaging modalities [27]. The crura of the diaphragm attach bilaterally to the vertebral column, defining their anatomical attachment zones. This study observed that the right crus is positioned anterolaterally to the vertebral bodies, while the left crus lies laterally. Most right crura attach to L1–L3, and left crura to L1–L2, consistent with prior anatomical studies [27, 28]. Notably, a subset of individuals exhibited higher-positioned diaphragmatic crura, originating from T12 (approximately 28.3% of cases), with only one case demonstrating a crus originating from L2, highlighting significant anatomical variability.

Previous studies have mainly concentrated on the measurement of the diameters of the aorta and veins, but there has been relatively little research on the relative positioning of the vessels to the thoracolumbar and lumbar vertebrae [29]. In this study, the overlapping area between the vessels and the vertebral body was measured as the vascular height. No major vessels should be injured intraoperatively; hence, there was no distinction made between the aorta and veins. When the majority of the vessels shifted anterior to the L1/L2 level, the vascular height was at its minimum. At L4-L5, multiple branches of the vessels appeared, resulting in the maximum vascular height. In the T10-T12 region, some individuals exhibited a leftward deviation of the vessels, which led to a larger vascular height and also showed certain anatomical variations.

Magnetic resonance imaging (MRI) studies by Hasegawa et al. [30] have demonstrated the longest nerve roots at the L5 level, while Regev et al. [22] reported the largest cross-sectional dimensions of nerve roots at the L4/L5 levels, respectively.The results demonstrated bilateral symmetry of the nerve roots. While previous studies have predominantly focused on the lateral height variation of lumbar nerve roots, our findings not only corroborate these morphological trends but also provide supplementary anatomical data regarding the nerve roots in the lower thoracic spine region.

Our findings demonstrated a progressive decrease in the safety area across lumbar levels, in agreement with previous reports [31]. Notably, at the L4/L5 level, the retroperitoneal vasculature exhibited posterior displacement while nerve roots adopted a more anterior positioning, collectively resulting in the narrowest safety area and the highest surgical risk. In contrast, the L1/L2 levels maintained the optimal safety profile. Furthermore, we identified a leftward deviation of the aorta in the lower thoracic region among a subset of subjects, correlating with elevated risk potential in corresponding anatomical zones. Due to the anatomical structures such as blood vessels, nerves, and muscles, the vertebral body and intervertebral disc resection of the lower thoracic and lumbar spine are typically approached laterally through the retroperitoneal extrapleural approach from the left side [32]. Preoperative measurements of the safe zone of adjacent intervertebral discs are taken using imaging studies, and surgery should be performed with the smaller level of the safe zone as the boundary to prevent damage to blood vessels and nerves, ensuring surgical safety.

Based on the aforementioned studies, preoperative CT evaluation facilitates delineation of safe operative zones surrounding the surgical site and quantitative assessment of vascular/neurological injury risks, thereby assisting spinal surgeons in preventing procedure-related complications.

## Conclusion

Three-dimensional CT reconstruction enables clear visualization of vascular structures, vertebral bodies, muscle attachment sites, nerve roots, and their spatial relationships. This modality further allows precise preoperative delineation of discectomy and corpectomy boundaries via transdiaphragmatic and psoas muscle approaches, while quantitatively assessing safety working zones for surgical planning.

## Supplementary Information

Below is the link to the electronic supplementary material.

Supplementary Material 1

Supplementary Material 2

Supplementary Material 3

Supplementary Material 4

Supplementary Material 5

Supplementary Material 6

## Data Availability

No datasets were generated or analysed during the current study.

## References

[1] Adkins DE, Sandhu FA, Voyadzis JM. Minimally invasive lateral approach to the thoracolumbar junction for corpectomy. J Clin Neurosci. 2013;20(9):1289–94. 10.1016/j.jocn.2012.09.051. 23830585 10.1016/j.jocn.2012.09.051

[2] Waddell WH, Gupta R, Stephens BF 2. nd. Thoracolumbar spine trauma. Orthop Clin North Am. 2021;52(4):481–9. 10.1016/j.ocl.2021.05.014. 10.1016/j.ocl.2021.05.014 34538355

[3] Denis F. The three column spine and its significance in the classification of acute thoracolumbar spinal injuries. Spine (Phila Pa 1976). 1983;8(8):817–31. 10.1097/00007632-198311000-00003. 6670016 10.1097/00007632-198311000-00003

[4] Naftchi AF, Vazquez S, Spirollari E, et al. Adult trauma patients with thoracolumbar injury classification and severity score of 4: A systematic review. Clin Spine Surg. 2023;36(6):237–42. 10.1097/BSD.0000000000001380. 35994034 10.1097/BSD.0000000000001380

[5] Harris EB, Sayadipour A, Massey P, Duplantier NL, Anderson DG. Mini-open versus open decompression and fusion for lumbar degenerative spondylolisthesis with stenosis. Am J Orthop (Belle Mead NJ). 2011;40(12):E257–61. 22268018

[6] Foley KT, Holly LT, Schwender JD. Minimally invasive lumbar fusion. Spine (Phila Pa 1976). 2003;28(15 Suppl):S26–35. 10.1097/01.BRS.0000076895.52418.5E. 12897471 10.1097/01.BRS.0000076895.52418.5E

[7] Zhang J, Liu TF, Shan H, et al. Decompression using minimally invasive surgery for lumbar spinal stenosis associated with degenerative spondylolisthesis: A review. Pain Ther. 2021;10(2):941–59. 10.1007/s40122-021-00293-6. 34322837 10.1007/s40122-021-00293-6 PMC8586290

[8] Walker CT, Xu DS, Godzik J, Turner JD, Uribe JS, Smith WD. Minimally invasive surgery for thoracolumbar spinal trauma. Ann Transl Med. 2018;6(6):102. 10.21037/atm.2018.02.10. 29707551 10.21037/atm.2018.02.10 PMC5900064

[9] Phan K, Rao PJ, Kam AC, Mobbs RJ. Minimally invasive versus open transforaminal lumbar interbody fusion for treatment of degenerative lumbar disease: systematic review and meta-analysis. Eur Spine J. 2015;24(5):1017–30. 10.1007/s00586-015-3903-4. 25813010 10.1007/s00586-015-3903-4

[10] Abbasi H, Miller L, Abbasi A, Orandi V, Khaghany K. Minimally invasive scoliosis surgery with oblique lateral lumbar interbody fusion: single surgeon feasibility study. Cureus. 2017;9(6):e1389. 10.7759/cureus.1389. Published 2017 Jun 25. 28775929 10.7759/cureus.1389 PMC5526703

[11] Ozgur BM, Aryan HE, Pimenta L, Taylor WR. Extreme lateral interbody fusion (XLIF): a novel surgical technique for anterior lumbar interbody fusion. Spine J. 2006;6(4):435–43. 10.1016/j.spinee.2005.08.012. 16825052 10.1016/j.spinee.2005.08.012

[12] Moller DJ, Slimack NP, Acosta FL Jr, Koski TR, Fessler RG, Liu JC. Minimally invasive lateral lumbar interbody fusion and Transpsoas approach-related morbidity. Neurosurg Focus. 2011;31(4):E4. 10.3171/2011.7.FOCUS11137. 21961867 10.3171/2011.7.FOCUS11137

[13] Uribe JS, Smith WD, Pimenta L, et al. Minimally invasive lateral approach for symptomatic thoracic disc herniation: initial multicenter clinical experience. J Neurosurg Spine. 2012;16(3):264–79. 10.3171/2011.10.SPINE11291. 22176427 10.3171/2011.10.SPINE11291

[14] Anand N, Baron EM, Thaiyananthan G, Khalsa K, Goldstein TB. Minimally invasive multilevel percutaneous correction and fusion for adult lumbar degenerative scoliosis: a technique and feasibility study. J Spinal Disord Tech. 2008;21(7):459–67. 10.1097/BSD.0b013e318167b06b. 18836355 10.1097/BSD.0b013e318167b06b

[15] Benglis DM, Elhammady MS, Levi AD, Vanni S. Minimally invasive anterolateral approaches for the treatment of back pain and adult degenerative deformity. Neurosurgery. 2008;63(3 Suppl):191–6. 10.1227/01.NEU.0000325487.49020.91. 18812924 10.1227/01.NEU.0000325487.49020.91

[16] Kepler CK, Bogner EA, Herzog RJ, Huang RC. Anatomy of the Psoas muscle and lumbar plexus with respect to the surgical approach for lateral Transpsoas interbody fusion. Eur Spine J. 2011;20(4):550–6. 10.1007/s00586-010-1593-5. 20938787 10.1007/s00586-010-1593-5 PMC3065600

[17] Beveridge TS, Fournier DE, Groh AMR, Johnson M, Power NE, Allman BL. The anatomy of the infrarenal lumbar splanchnic nerves in human cadavers: implications for retroperitoneal nerve-sparing surgery. J Anat. 2018;232(1):124–33. 10.1111/joa.12721. 29159805 10.1111/joa.12721 PMC5735059

[18] Regev GJ, Lee YP, Taylor WR, Garfin SR, Kim CW. Nerve injury to the posterior Rami medial branch during the insertion of pedicle screws: comparison of mini-open versus percutaneous pedicle screw insertion techniques. Spine (Phila Pa 1976). 2009;34(11):1239–42. 10.1097/BRS.0b013e31819e2c5c. 19444073 10.1097/BRS.0b013e31819e2c5c

[19] Costanzo G, Zoccali C, Maykowski P, Walter CM, Skoch J, Baaj AA. The role of minimally invasive lateral lumbar interbody fusion in sagittal balance correction and spinal deformity. Eur Spine J. 2014;23(Suppl 6):699–704. 10.1007/s00586-014-3561-y. 25217242 10.1007/s00586-014-3561-y

[20] Dakwar E, Cardona RF, Smith DA, Uribe JS. Early outcomes and safety of the minimally invasive, lateral retroperitoneal Transpsoas approach for adult degenerative scoliosis. Neurosurg Focus. 2010;28(3):E8. 10.3171/2010.1.FOCUS09282. 20192668 10.3171/2010.1.FOCUS09282

[21] Gu Y, Ebraheim NA, Xu R, Rezcallah AT, Yeasting RA. Anatomic considerations of the posterolateral lumbar disk region. Orthopedics. 2001;24(1):56–8. 10.3928/0147-7447-20010101-20. 11199353 10.3928/0147-7447-20010101-20

[22] Regev GJ, Chen L, Dhawan M, Lee YP, Garfin SR, Kim CW. Morphometric analysis of the ventral nerve roots and retroperitoneal vessels with respect to the minimally invasive lateral approach in normal and deformed spines. Spine (Phila Pa 1976). 2009;34(12):1330–5. 10.1097/BRS.0b013e3181a029e1. 19455010 10.1097/BRS.0b013e3181a029e1

[23] Christiansen PA, Huang S, Smith JS, Shaffrey ME, Uribe JS, Yen CP. Mini-open lateral retropleural/retroperitoneal approaches for thoracic and thoracolumbar junction anterior column pathologies. Neurosurg Focus. 2020;49(3):E13. 10.3171/2020.6.FOCUS20360. 32871570 10.3171/2020.6.FOCUS20360

[24] Moro T, Kikuchi S, Konno S, Yaginuma H. An anatomic study of the lumbar plexus with respect to retroperitoneal endoscopic surgery. Spine (Phila Pa 1976). 2003;28(5):423–8. 10.1097/01.BRS.0000049226.87064.3B. 12616150 10.1097/01.BRS.0000049226.87064.3B

[25] Fantini GA, Pappou IP, Girardi FP, Sandhu HS, Cammisa FP Jr. Major vascular injury during anterior lumbar spinal surgery: incidence, risk factors, and management. Spine (Phila Pa 1976). 2007;32(24):2751–8. 10.1097/BRS.0b013e31815a996e. 18007256 10.1097/BRS.0b013e31815a996e

[26] Kuklo TR, Lehman RA Jr, Lenke LG. Structures at risk following anterior instrumented spinal fusion for thoracic adolescent idiopathic scoliosis. J Spinal Disord Tech. 2005;18:S58–64. 10.1097/01.bsd.0000123424.12852.75. 15699806 10.1097/01.bsd.0000123424.12852.75

[27] Dakwar E, Ahmadian A, Uribe JS. The anatomical relationship of the diaphragm to the thoracolumbar junction during the minimally invasive lateral extracoelomic (retropleural/retroperitoneal) approach. J Neurosurg Spine. 2012;16(4):359–64. 10.3171/2011.12.SPINE11626. 22225484 10.3171/2011.12.SPINE11626

[28] Baaj AA, Papadimitriou K, Amin AG, Kretzer RM, Wolinsky JP, Gokaslan ZL. Surgical anatomy of the diaphragm in the anterolateral approach to the spine: a cadaveric study. J Spinal Disord Tech. 2014;27(4):220–3. 10.1097/BSD.0b013e3182a18125. 24869984 10.1097/BSD.0b013e3182a18125

[29] Kot A, Polak J, Klepinowski T, et al. Morphometric analysis of the lumbar vertebrae and intervertebral discs in relation to abdominal aorta: CT-based study. Surg Radiol Anat. 2022;44(3):431–41. 10.1007/s00276-021-02865-9. 34874459 10.1007/s00276-021-02865-9 PMC8917002

[30] Hasegawa T, Mikawa Y, Watanabe R, An HS. Morphometric analysis of the lumbosacral nerve roots and dorsal root ganglia by magnetic resonance imaging. Spine (Phila Pa 1976). 1996;21(9):1005–9. 10.1097/00007632-199605010-00001. 8724082 10.1097/00007632-199605010-00001

[31] Guérin P, Obeid I, Gille O, et al. Safe working zones using the minimally invasive lateral retroperitoneal Transpsoas approach: a morphometric study. Surg Radiol Anat. 2011;33(8):665–71. 10.1007/s00276-011-0798-6. 21384202 10.1007/s00276-011-0798-6

[32] Litré CF, Duntze J, Benhima Y, et al. Anterior minimally invasive extrapleural retroperitoneal approach to the thoraco-lumbar junction of the spine. Orthop Traumatol Surg Res. 2013;99(1):94–8. 10.1016/j.otsr.2012.08.006. 23246007 10.1016/j.otsr.2012.08.006

